# Connexin 31.1 degradation requires the Clathrin-mediated autophagy in NSCLC cell H1299

**DOI:** 10.1111/jcmm.12470

**Published:** 2014-11-11

**Authors:** Xingli Zhu, Zhenchao Ruan, Xiufang Yang, Kaili Chu, Hai Wu, Yao Li, Yan Huang

**Affiliations:** State Key Laboratory of Genetic Engineering, Institute of Genetics, School of Life Sciences, Fudan UniversityShanghai, China

**Keywords:** Connexin 31.1, ubiquitin–proteasome system, starvation, autophagy, clathrin

## Abstract

Connexins have relative short half-lives. Connexin 31.1 (Cx31.1) was newly reported to be down-regulated in non-small cell lung cancer cell lines, and displayed tumour-suppressive properties. However, no reports describing how a cell regulates Cx31.1 level were found. In this study, Cx31.1 was suggested to be degraded through both ubiquitin–proteasome system (UPS) and autophagy. Blockage of UPS with MG-132 increased Cx31.1 level, but could not inhibit the degradation of Cx31.1 completely. In H1299 cells stably expressing Cx31.1, Cx31.1 reduced when autophagy was induced through starvation or Brefeldin A treatment. Knockdown of autophagy-related protein ATG5 could increase the cellular level of Cx31.1 both under normal growth condition and starvation-induced autophagy. Colocalization of Cx31.1 and autophagy marker light chain 3 (LC3) was revealed by immunofluorescence analysis. Coimmunoprecipitation and immunofluorescence showed that Cx31.1 might interact with clathrin heavy chain which was newly reported to regulate autophagic lysosome reformation (ALR) and controls lysosome homoeostasis. When clathrin expression was knockdown by siRNA treatment, the level of Cx31.1 increased prominently both under normal growth condition and starvation-induced autophagy. Under starvation-induced autophagy, LC3-II levels were slightly accumulated with siCla. treatment compared to that of siNC, which could be ascribed to that clathrin knockdown impaired the late stage of autophagy, ALR. Taken together, we found autophagy contributed to Cx31.1 degradation, and clathrin might be involved in the autophagy of Cx31.1.

## Introduction

Vertebrate gap junctions, composed of integral membrane proteins encoded by the connexin gene family, are critically important in regulation of embryonic development, co-ordinated contraction of excitable cells, tissue homoeostasis, normal cell growth and differentiation. Connexins are prominent in their short half-lives, which are about 1.5–5 hrs. Both the ubiquitin-proteasomal and endo-lysosomal pathways have been implicated in connexin turnover [1].

Recently, autophagy emerged as a new mechanism for connexin degradation. In Cx43-GFP-expressing HeLa cells, endocytosed gap junctions were reported to be degraded by autophagy independent of starvation [2]. Autophagy may contribute to endogenously and exogenously expressed wild-type Cx43 and Cx50 proteins in both un-induced and starvation-induced cells [3].

Clathrin is a trimeric assembly consisting of three heavy chains, each with an associated light chain (LC) [4]. In non-dividing cells, clathrin forms coats on membranes destined for vesicular transport either from the plasma membrane to endosomes or between endosomes and trans-Golgi network [5]. Recent study demonstrated that clathrin participated in regulating autophagic lysosome reformation (ALR) when autophagy happened [6].

Connexin 31.1 (also known as GJB5) rarely forms functional gap junction channels, either with itself or other connexin isoforms [7]. It displayed anti-tumour effect in H1299 cells according to our previous analysis [8]. The expression of Cx31.1 was reversely correlated with the metastasis potential in non-small cell lung cancer (NSCLC) cell lines. To maintain the balance of Cx31.1, a protein from an active family, an efficient degradation mechanism is necessary to ensure the dynamic turnover of Cx31.1. Therefore, we focused on degradation mechanisms of Cx31.1, which may help us to understand why Cx31.1 was poorly expressed in malignant NSCLC cells.

Our present data revealed that in H1299 cell, Cx31.1 has a short half-life of only 1–2 hrs; both autophagy and proteasomal pathway are involved in Cx31.1 degradation. Moreover, clathrin may interact with Cx31.1 to mediate Cx31.1 autophagy.

## Materials and methods

### DNA constructs

The Cx31.1-EGFP expressing construct was the same one as used in our previous work [8]. The plasmid mCherry-LC3 was purchased from Yrbio Co.Ltd (Changsha, China).

### Cell culture and plasmid transfection

H1299 cells were obtained from American Type Culture Collection (Manassas, VA, USA). All cells were maintained in RPMI Medium 1640 (Gibco, Eggenstein, Germany) supplemented with 10% foetal bovine serum (Invitrogen, Carlsbad, CA, USA), 1% penicillin and streptomycin (Gibco) and 1 mM sodium pyruvate (Gibco) at 37°C and 5% CO_2_ in a humidified incubator. H1299 cells stably expressing Cx31.1-EGFP (Cx31.1-EGFP-H1299 cells) or EGFP were the same as previous [8].

### Cell treatment

To analyse half-life of Cx31.1, the culture growth medium was replaced with normal growth medium containing 20 μg/ml cycloheximide (CHX). To inhibit proteasomal degradation of Cx31.1, cells were treated with normal growth media containing 50 μM MG-132 for 6 hrs. Cells were treated with both 20 μg/ml CHX and 50 μM MG-132 for 1 or 2 hrs to further indicate the degradation pathway of Cx31.1. In starvation treatment, cells were grown to about 70–80% confluence. Then the growth medium was replaced with Hanks balanced salt solution (HBSS, Gibco) for starved cells or normal growth medium for control cells. The cells were then incubated in HBSS for 2 or 4 hrs. To block ER–Golgi transportation, Brefeldin A (BFA) was employed and cells were treated with normal growth medium containing 10 μg/ml BFA for 24 hrs. For chloroquine treatment, cells were grown to about 70–80% confluence. Then, the culture growth medium was replaced with normal growth medium containing 30 μM chloroquine for 6 hrs. In the case of starvation, the cells were first incubated with CLQ-containing growth medium for 4 hrs or 2 hrs, rinsed twice with HBSS, and then the growth media was replaced with HBSS containing 30 μM chloroquine to starve cells for 2 or 4 hrs, respectively.

### siRNA transfection

According to previous reports, clathrin heavy chain and ATG5 can be knockdown using siRNA target sequences as follows: siCla.-1: GCUGGGAAAACUCUUCAGA, siCla.-2: UAAUCCAAUUCGAAGACCAAU [9]; siAtg5-1 GCAACTCTGGATGGGATTG, siAtg5-2 CATCTGAGCTACCCGGATA [10]. A siRNA lacks homology to the genome was employed as a negative control. They were chemically synthesized by GenePharma (Shanghai, China). The silencing efficiencies were evaluated by Western blotting 48 hrs after siRNAs or siNC transfection.

### Immunofluorescence and fluorescent visualization

Cells were grown on coverslips (Thermo Fisher Scientific, Rochester, NY, USA) and washed, fixed in 4% paraformaldehyde for 15 min. at 4°C, and blocked in 5% normal gelatin diluted in PBS with 0.3% Triton X-100. After a brief wash in PBS, cells were incubated with primary antibodies (anti-Golgi 58k, 1:100, Sigma-Aldrich, St Louis, MO, USA; anti-PSMA2, 1:100, Cell Signaling Technology, Beverly, MA, USA) overnight at 4°C. The secondary antibodies conjugated with DyLight (KPL, Gaithersburg, MD, USA) were used to detect primary antibodies. Organelle Lights endosomes-RFP (Invitrogen) was employed to visualize endosome of Cx31.1-EGFP-H1299 cells according to the manual of the manufacture. To visualize the colocalization of mCherry-LC3 and Cx31.1-EGFP, Cx31.1-EGFP-H1299 cells were transiently transfected with mCherry-LC3. The cells were then observed under a Nikon ECLIPSE research microscope (Nikon, Tokyo, Japan).

As to the immunofluorescence of clathrin, we fixed the cells in methanol at 4°C for 10 min., and the anti-clathrin heavy chain (1:100, Cell Signaling Technology) was used for detection. Fluorescence signals were observed under the ZEISS LSM710 (Zeiss, Oberkochen, Germany) spectral confocal microscopy.

### Immunoprecipitation

Briefly, cell lysates were prepared using immunoprecipitation assay buffer [50 mM Tris-HCl (pH 7.4), 150 mM sodium chloride, 1% Nonidet P-40, 0.25% sodium deoxycholate, 1 mM EDTA, 1 mM sodium fluoride, 1 mM Sodium Orthovanadate] supplemented with protease inhibitors (100 μg/ml phenylmethylsulfonyl fluoride (PMSF), Sigma-Aldrich; Complete Protease Inhibitor Cocktail, Roche, Indianapolis, IN, USA), then incubated with rabbit IgG together with 20 μl resuspended protein A/G PLUS-Agarose (Santa Cruz Biotechnology, Santa Cruz, CA, USA) for 30 min. at 4°C. The supernatants were incubated with polyclonal anti-GFP (Abcam, Cambridge, MA, USA) antibody with shaking for 1 hr at 4°C, followed by overnight incubation with protein A/G PLUS-Agarose beads. The beads were then washed vigorously with immunoprecipitation assay buffer for four times. The eluted samples were then separated by SDS-PAGE, and subjected to high-performance liquid chromatography-tandem mass spectrometry (HPLC-MS/MS) analysis, as well as immunoblotting to detect clathrin heavy chain.

Reciprocally, we also used primary antibody anti-clathrin heavy chain for immunoprecipitation. Samples were then separated by SDS-PAGE and immunoblotted with primary antibody against GFP (NeoMarkers, Fremont, CA, USA).

### Protein identification through HPLC-MS/MS

Protein bands in PAGE gel were subjected to in-gel tryptic digestion using standard procedures [11]. To avoid interference between gel slices, especially for those degraded fragments, in the courses of LC separation and MS detection, the digested peptides were analysed from low MWs to high MWs. The sample treatment, HPLC–MS analysis was the same as reported previously [12].

### Immunoblotting

Cell lysates were prepared using radio immunoprecipitation assay buffer supplemented with protease inhibitors, separated by electrophoresis, transferred onto polyvinylidenedifluoride membranes. Membranes were blocked with 5% non-fat milk in TBS containing 0.05% Tween-20, and incubated overnight at 4°C with primary antibodies (GFP, 1:2000, NeoMarkers; ATG5, 1:1000; LC3, 1:1000, Protein Tech Group, Wuhan, China; Clathrin, 1:1000, Cell Signaling Technology; β-tubulin, 1:3000, Sigma-Aldrich). The blots were washed, and incubated in peroxidase-coupled secondary antibodies against rabbit or mouse IgG (1:5000, KPL) at room temperature, washed, and developed using Super Signal Substrate (Pierce, Rockford, IL, USA). Signal intensity of Western blot was quantified with Quantity One Software (Bio-Rad, Richmond, CA, USA).

## Results

### Cx31.1 has a short life in H1299 cells

**Fig. 1:**
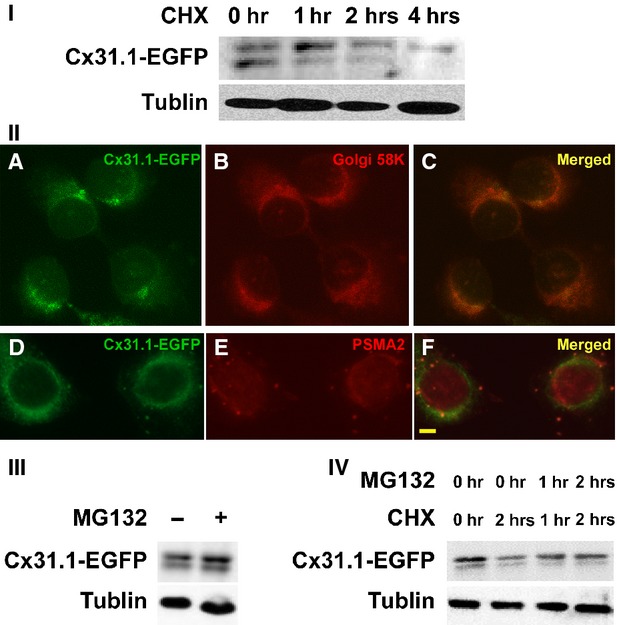
Cx31.1 could be degraded by UPS. (**I**) Half-life analysis of Cx31.1 in Cx31.1-EGFP-H1299 cells by cycloheximide (CHX) treatment. Western blot revealed that level of Cx31.1-EGFP was reduced more than a half after treated with CHX for 1–2 hrs. (**II**) Ectopically expressed Cx31.1-EGFP protein was observed to be colocalized with Golgi (C), and proteasome (F) marker protein 58K and PSMA2 respectively. Scale bar, 10 μm. Note that PSMA2 also showed immunofluorescence in the nuclei, as indicated in the manufacturer's instruction. (**III**) MG-132 was used to inhibit proteasome activity for blocking UPS. Blocking UPS increased level of Cx31.1-EGFP in H1299 cells under normal growth condition. (**IV**) Levels of Cx31.1-EGFP in cells treated with CHX alone or CHX plus MG-132. Note that when the cells were treated by CHX for 2 hrs, MG132 could increase the amount of Cx31.1-EGFP compared to no MG-132 treatment.

Proteins are continuously synthesized and degraded in the life of all cells to maintain a proper amount for its function. The half-life of connexin family member's is only 1.5–5 hrs [1]. According to our analysis, Cx31.1-EGFP is a protein with a half-life of 1–2 hrs in cultured H1299 cells as revealed by CHX treatment (Fig. 1I). It suggested that Cx31.1 has a high turnover rate.

Among connexin family members, Cx43 also has a half-life of only 1–2 hrs in cultured cell [13]. Lysosomes and proteasomes are both involved in Cx43 degradation [14]. Thus, an effective degradation system might as well be necessary for Cx31.1.

### Intracellular localization of Cx31.1 ectopically expressed in H1299 cells

Previous studies identified proteasomal, endo-lysosomal and phago-lysosomal degradation pathways in the regulation of connexin protein degradation and gap junction stability [1,14–19]. However, it was reported that Cx31.1 does not form heterotypic or homotypic gap junction channels [7]. To determine the degradation pathway of Cx31.1 in H1299 cells, we first investigated intracellular localization of Cx31.1. Our previous data indicated that Cx31.1-EGFP showed colocalization with endoplasmic reticulum marker GRP94 and lysosome marker LAMP1 [8]. To further interpret the cellular localization of Cx31.1, we immunostained Cx31.1-EGFP-H1299 cells with antibody against Golgi 58KD (Marker of Golgi) and PSMA2 (Marker of Proteasome), as well as transfection with organelle lights endosomes-RFP (Invitrogen). As shown in Figure 1II, some of Cx31.1-EGFP colocalized with Golgi 58KD and PSMA2. However, we had not found Cx31.1 in endosome (data not shown). It should be pointed out that PMSA2 also showed immunofluorescence in the nuclei, as indicated in the manufacturer's instruction.

Thus, Cx31.1 could be localized to ER, Golgi, proteasome and lysosome, but did not localize to endosome. As both lysosomes and autolysosomes express LAMP1, we speculated that among the three possible ways participating in the degradation of connexin family proteins, Cx31.1 may choose either proteasomal pathway or phago-lysosomal pathway.

### Role of proteasomal degradation in life cycle of Cx31.1

According to the cellular localization of ectopically expressed Cx31.1, we first considered the role of the ubiquitin–proteasome system in Cx31.1 degradation. Cx31.1-EGFP-H1299 cells were treated with proteasomal inhibitor MG-132. Under normal growth condition, Cx31.1-EGFP increased 30% in cells treated with MG-132 compared with control cells (Fig. 1III). When the protein synthesis was inhibited by CHX for 2 hrs, Cx31.1-EGFP level in cells treated for 2 hrs with MG-132 increased 48% compared to that without MG-132 (Fig. 1IV). Importantly, the inhibition of Cx31.1 degradation was not completely blocked by MG-132 treatment, as the Cx31.1-EGFP level in cells treated with both CHX and MG-132 was still much less than those in normal growth medium (Fig. 1IV). These results, together with the information of Cx31.1 cellular localization, suggested that autophagy might be as another way for Cx31.1 degradation in H1299 cells.

### Starvation- and BFA-induced autophagy decreases levels of Cx31.1

**Fig. 2:**
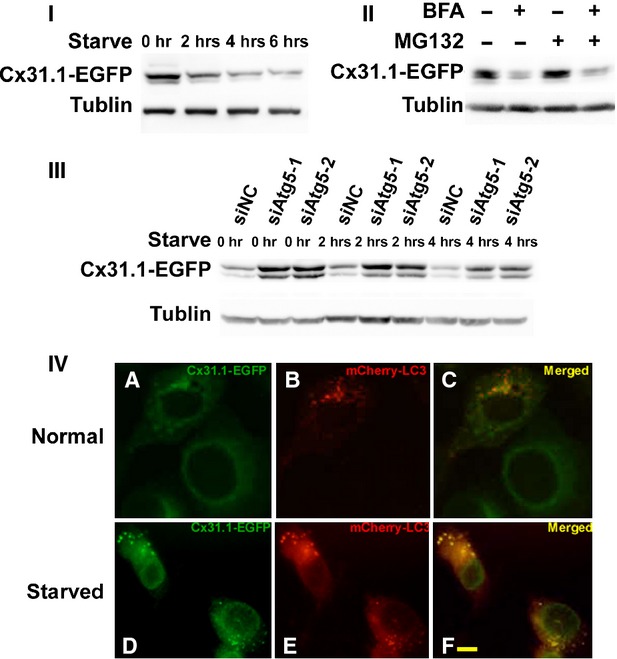
Cx31.1 could be degraded by autophagy. (**I**) Autophagy induction through starvation gradually decreased the level of Cx31.1 at 2 h, 4 h and 6 h in H1299 cells stably expressing Cx31.1-EGFP. (**II**) Autophagy induction by Brefeldin A treatment decreased Cx31.1-EGFP level, both with or without MG-132. (**III**) Autophagy inhibition by ATG5 siRNAs treatment increased Cx31.1-EGFP levels, both under normal growth condition and starvation. (**IV**) Cx31.1-EGFP colocalizes with autophagosome marker LC3. These were images of H1299-Cx31.1-EGFP cells transiently transfected with a DNA construct coding for mCherry-LC3 (red). Overlapped puncta had been shown under normal growth condition, as well as under starvation. Scale bar, 10 μm.

Starvation is one of the ways to induce autophagy. We starved Cx31.1-EGFP-H1299 cells and found a time-dependent decrease in Cx31.1-EGFP levels compared with those incubated under normal growth conditions. As shown in Figure 2I, levels of Cx31.1-EGFP decreased about 54%, 33% and 29% at 2, 4 and 6 hrs of starvation respectively.

We showed that Cx31.1 localized to ER [8], as well as to Golgi (Fig. 1II). Brefeldin A is an inhibitor of vesicle transport between ER and Golgi, and autophagy can also be induced by Brefeldin A that causes ER stress [20]. We compared levels of Cx31.1-EGFP in cells treated with or without Brefeldin A. Cx31.1-EGFP level decreased to 32% had been shown in Brefeldin A treatment compared to cells grew in normal condition (Fig. 2II). As proteins accumulated in ER may be degraded by the 26S proteasome through ER-association protein degradation, we also used proteasomal inhibitor MG-132 to indicate the effect of Brefeldin A. We found that level of Cx31.1-EGFP in cells treated with both MG-132 and BFA was 26% of that in cells treated with MG-132 alone. These analyses suggested that autophagy induction by starvation and BFA might influence Cx31.1 level in H1299 cells.

### ATG5 knockdown increased Cx31.1-EGFP

Autophagy depends on the function of several autophagy-related (Atg) proteins. We genetically inactivated this process using siRNA against ATG5, an ubiquitin-like protein essential for autophagosome expansion and completion [21] to examine the autophagy-mediated degradation of Cx31.1. We synthesized and transfected ATG5 siRNA into Cx31.1-EGFP-H1299 cells. Both of the two siRNAs showed silencing effect to down-regulate Atg5 protein expression as confirmed by Western blots (Fig. S1). Under normal growth conditions, knockdown of Atg5 increased the level of Cx31.1-EGFP about threefold compared with that of controls. When the cells were starved for 2 and 4 hrs, knockdown of Atg5 also increased the level of Cx31.1-EGFP about threefold compared with that of controls. Meanwhile, Cx31.1-EGFP levels were gradually decreased from normal growth condition to 2 hrs starvation and 4 hrs starvation, both under siNC treatment and siATG5 treatment. These results implicated the participation of Atg5-dependent autophagy in the starvation-induced degradation of Cx31.1.

### Cx31.1 could colocalize with autophagosomal marker LC3

In autophagy system, the microtubule-associated protein LC3 is a mammalian homologue of yeast autophagosomal membrane component and is regarded as the most specific autophagosomal marker [22]. If autophagy participates in degradation of Cx31.1, Cx31.1 should colocalize with autophagosomal marker LC3. To test this hypothesis, H1299-Cx31.1-EGFP cells were transfected with plasmid mCherry-LC3. Under control conditions, mCherry-LC3-labelled vesicular structures were observed in the cytoplasm of transfected cells, and Cx31.1-EGFP fluorescence were occasionally observed to associate with mCherry-LC3 fluorescence, suggesting degradation of Cx31.1-EGFP by constitutive autophagy (Fig. 2IV). When H1299-Cx31.1-EGFP cells were starved for 2 hrs, several of the intracellular Cx31.1-EGFP-containing puncta were also labelled with mCherry-LC3 (Fig. 2IV). The colocalization of Cx31.1-EGFP and autophagosomal marker LC3 further supported that Cx31.1-EGFP could be degraded by autophagy.

### Clathrin might interact with Cx31.1-EGFP

**Fig. 3:**
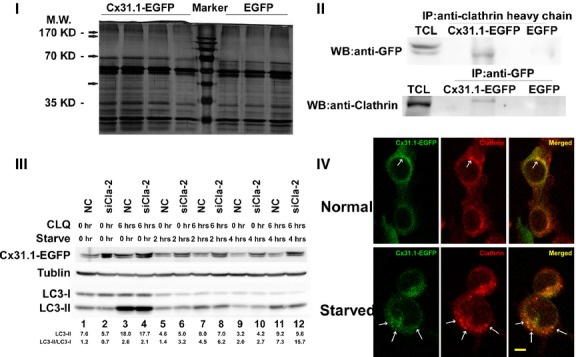
Cx31.1 could interact with clathrin, and clathrin might involve in the autophagy of Cx31.1-EGFP. (**I**) Characterization of proteins coimmunoprecipitated with Cx31.1. Silver-stained gel of protein after immunoprecipitation with anti-GFP. Bands of probable interactors of Cx31.1 were marked with arrows. H1299 cells stably expressing EGFP were used as a control. These bands were excised, in-gel digested and analysed by HPLC-MS on an LTQ-FT instrument using two consecutive stages of tandem MS. This experiment was repeated for three times. (**II**) Cx31.1-EGFP was coimmunoprecipitated together with clathrin. TCL, total cell lysate; Cx31.1-EGFP, protein coimmunoprecipitated from Cx31.1-EGFP-H1299 cells; EGFP, protein coimmunoprecipitated from H1299 cells stably expressing EGFP. The samples were coimmunoprecipitated with antibody against clathrin heavy chain, then blotted with antibody against GFP and vice versa. (**III**) Knockdown clathrin expression enhanced Cx31.1-EGFP levels. Cx31.1-EGFP-H1299 cells were treated with siCla-2 or in combination with CLQ under normal growth condition, as well as starved for 2 and 4 hrs. Immunoblots were performed with antibodies against GFP and LC3. Cx31.1-EGFP-H1299 cells were transiently transfected with siCla-2 for 48 hrs. Before the cells lysates were collected, cells were treated with or without 30 μM CLQ for 6 hrs. In the case of starvation, the cells were incubated with CLQ-containing growth medium for 4 hrs or 2 hrs, and then the growth media was replaced with HBSS containing 30 μM chloroquine to starve cells for 2 or 4 hrs respectively. Cx31.1-EGFP levels were enhanced after siCla-2 treatment with or without serum starvation. LC3 was used as an indicator for autophagy. Duplicated gel was made for the immunoblotting of β-tubulin. The relative amount of LC3-II and the ratio of LC3-II/LC3-I were indicated. (**IV**) Confocal images of colocalization of Cx31.1-EGFP and clathrin under normal growth condition and starvation. Arrows indicated puncta with both Cx31.1-EGFP fluorescence and anti-clathrin immunoreactivities. Scale bar, 10 μm.

We performed mass spectrometry analysis on cell lysates immunoprecipitated with an antibody against GFP to obtain proteins interacted with Cx31.1 in Cx31.1-EGFP-H1299 cells. H1299 cells stably expressing EGFP were used as control. Protein mixture immunoprecipitated together with Cx31.1-EGFP or EGFP were separated on SDS-PAGE. Silver-stained gels were showed in Figure 3I. Bands of probable interactors of Cx31.1-EGFP which were not appeared in the control were marked with arrows. We characterized proteins in those bands by HPLC-MS/MS after in-gel trypsinization. Among the identified proteins which may potentially interact with Cx31.1, clathrin heavy chain (a major component of coated vesicles that has a vital function in the endocytic pathway) was found several times in each of the three repeated tests.

We then validated the interaction between Cx31.1-EGFP and clathrin heavy chain by performing coimmunoprecipitation experiment using antibody against clathrin as bait. The cell lysate was immunoprecipitated with antibody anti-GFP, then immunoblotted with antibody against clathrin heavy chain. Reciprocally, the cell lysate was immunoprecipitated against clathrin heavy chain, then immunoblotted with antibody against GFP. As shown in Figure 3II, clathrin could be coisolated along with Cx31.1-EGFP as a binding partner in Cx31.1-EGFP-H1299 cells and vice versa; while in H1299 cells stably expressing EGFP, clathrin was not immunoprecipitated. The results indicated clathrin could interact with Cx31.1.

To further confirm the interaction between clathrin and Cx31.1, we tested the colocalization of Cx31.1-EGFP and clathrin with or without cell starvation. As shown in Figure 3IV, under normal growth condition, the immunofluorescence of clathrin could be found throughout the entire cytoplasm with concentrated patches in perinuclear regions, as well as discrete puncta. The staining pattern was in consistency with previous report [23]. Cx31.1-EGFP also showed patched perinuclear and punctated fluorescent in the cytoplasm. Under normal growth condition, punctum with both Cx31.1-EGFP and clathrin staining was marked with arrow. We then analysed the localization of Cx31.1-EGFP and clathrin when cells were starved for 2 hrs. As starvation can cause cells to round up, to rule out the possibility that colocalization was caused by starvation-induced shape change, we only focused on the puncta but not patches to reveal the colocalization of Cx31.1-EGFP and clathrin. We found that under starvation, the colocalized puncta were found more distinctive compared to that of the normal growth condition. The results further suggested the interaction of Cx31.1 and clathrin.

### Clathrin might be involved in the autophagy degradation of Cx31.1

Clathrin plays an important role in vesicular trafficking and mediates budding in various membrane systems [24]. Recently, Rong *et al*. reported the discovery of ALR, a process essential for lysosome homoeostasis following starvation-induced autophagy. Clathrin is a central component in ALR that can be recruited to autolysosomes [6].

The role of clathrin in autophagy, and its colocalization with Cx31.1-EGFP prompted us to speculate that clathrin might be required for the autophagy of Cx31.1-EGFP. To test this idea, we used siRNA to knockdown clathrin expression, and then examined the level of Cx31.1-EGFP, as well as the conversion of LC3-I to LC3-II. We found that the siRNA against clathrin (siCla-2) could efficiently knockdown clathrin expression in H1299 cells (Fig. S1). Thus, we used this siRNA for the following experiments.

Because lysosomes are involved in autophagy-mediated protein degradation, we first tested whether the degradation of Cx31.1-EGFP was dependent on the activity of lysosomal protease. As shown in the Figure S2, when H1299-Cx31.1-EGFP cells were treated with the lysosomal protease inhibitor, chloroquine (CLQ), the levels of Cx31.1-EGFP were increased under both normal growth condition and starvation-induced autophagy. Meanwhile, CLQ treatment caused LC3-II accumulation.

We then used siCla-2, as well as CLQ to treat H1299-Cx31.1-EGFP cells. When the cells were treated with siCla-2 alone, Cx31.1-EGFP was increased under normal growth condition (starve 0 hr). When the cells were starved for 2 and 4 hrs after siCla-2 treatment, the Cx31.1-EGFP levels also increased prominently compared to that of the control siRNA (Fig. 3III). Meanwhile, the expression of Cx31.1-EGFP levels was gradually decreased from normal growth condition to 2 hrs starvation and 4 hrs starvation, both under siNC treatment and siCla-2 treatment. In consistency with the Figure S2, the CLQ treatment under siNC transfection also led to the increase in Cx31.1-EGFP (compare lane 1 and 3, *etc*.). When cells were treated with both CLQ and siCla-2, the level of Cx31.1-EGFP further increased compared to that of siCla-2 alone (compare lane 2 and 4, *etc*.).

The detection of LC3 conversion (LC3-I to LC3-II) is a widely used approach to monitor autophagy because the amount of LC3-II is clearly correlated with the number of autophagosomes. However, it would be more appropriate to compare the amount of LC3-II between samples [25].

We found that the ratio of LC3-II/LC3-I increased when cells were treated with siCla-2 (Fig. 3III) under starvation. The combination of CLQ and siCla-2 treatment led to a more significant increase in LC3-II/LC3-I change (compare lane 6 and 8; lane 10 and 12). As shown in Figure 3III, under starvation-induced autophagy, there was a slight increase in the LC3-II level (compare lane 5 and 6; lane 9 and 10) when cells were treated with siCla-2. Overall, the accumulation of LC3-II at 2 and 4 hrs starvation was weak.

It had been pointed out that if the LC3-II level were to remain unchanged, it is likely that autophagosome accumulation occurred because of inhibition of autophagic degradation [25]. As clathrin was essential to ALR, clathrin knockdown would impair this late stage of autophagy. Taken together the function of clathrin in ALR, the amount change in LC3-II and Cx31.1-EGFP among different treatments, we speculated that clathrin was required for both basal and starvation-induced autophagy of Cx31.1-EGFP.

## Discussion

It is an intriguing question that connexins harbour highly dynamic turnover, which are exceedingly short compared to typical integral plasma membrane proteins. Defects in any of these connexin life stages might lead to disease or be associated with disease [26]. In recent years, advances have been made in the understanding of the pathways on connexin assembly, dynamics and degradation. Most of the previous study of connexin turnover focused on the ubiquitin-proteasomal system [14,27,28]. Recently, autophagic pathway for connexin protein degradation was also interpreted [2,3].

However, we know little about the degradation of Cx31.1, a connexin gene which may act as a tumour suppressor in NSCLC cells. Our current data suggest the contribution of proteasomal degradation, as well as ATG5-dependent autophagy to constitutive and starvation-induced degradation of Cx31.1 in H1299 cells. Coimmunoprecipitation and immunofluorescence showed that Cx31.1 might interact with clathrin heavy chain. During autophagy degradation pathway, clathrin acts as a central part to regulate ALR, a cellular mechanism required for maintaining lysosome homoeostasis during and after autophagy [6]. In our study, knockdown of clathrin expression could enhance the level of Cx31.1 both under normal growth condition and starvation. Cells usually go under basal, constitutive level of autophagy which plays an important role in cellular homoeostasis through the elimination of damaged/old organelles as well as the turnover of proteins, and thus maintains quality control of essential cellular components. When the basal level of autophagy is inhibited, cellular proteins may accumulate. In Atg5−/− cells, diffuse, abnormal intracellular proteins accumulate, and then form aggregates and inclusions [29].

However, Cx31.1 is not the only connexin binds to clathrin. When connexins form gap junction on the cell membrane between closely apposed cells, clathrin would localize to the gap junction plagues. For example, Gumpert *et al*. reported that gap junction internalization is a clathrin-mediated endocytic process that utilizes the vesicle-coat protein clathrin in Cx43-GFP-transfected HeLa cells [30]; Piehl *et al*. reported that gap junction plaques can be internalized to form large, double-membrane vesicles by clathrin-dependent endocytosis machinery components [31]. However, these papers are regarding the endocytosis of Cx43 gap junctions by clathrin-dependent pathways.

It had been reported that clathrin also plays a role in autophagosome formation. In mammalian cells, the heavy chain of clathrin interacts with Atg16L1 and is involved in the formation of Atg16L1-positive early autophagosome precursors [32]. However, to address clathrin functions in autophagosome formation, clathrin knockdown efficiency needs to be extremely high for it to affect autophagosome formation. Rong *et al*. pointed out that when they examine the ALR, clathrin knockdown efficiency is moderate. They inferred that moderate clathrin knockdown efficiency allows for autophagosome formation and progression to the autolysosome stage; whereas a highly effective clathrin knockdown blocks the formation of autophagosomes [6].

During autophagy, lysosomes fuse with an autophagosome to form an autolysosome, in which digestion occurs. Endogenous LC3 is detected as two bands following SDS-PAGE and immunoblotting. That is, LC3-I, which is cytosolic; and LC3-II, which is conjugated with phosphatidylethanolamine and is present on isolation membranes and autophagosomes. During a short starvation period, the amount of LC3-I decreases and that of LC3-II increases. However, if cells are subjected to longer starvation, both LC3-I and LC3-II would be degraded [33]. Compare the amount of LC3-II between samples is more appropriate to indicate autophagy flux [25]. In our study, when Cx31.1-EGFP-H1299 cells transfected with siCla-2 starved for 2 and 4 hrs, we can observe a slight accumulation of the amount of LC3-II as siCla-2 blocked the autophagosome–lysosome fusion, and the digestion was delayed. This is similar to that of inhibition of autophagic degradation, that is, LC3-II level remains unchanged because of autophagosome accumulation [25].

We previously reported that Cx31.1 displayed tumour-suppressive properties, and its expression was negatively related to the metastasis potential in NSCLC cell lines [8]. Clathrin-mediated autophagy of Cx31.1 provided us information to understand why the expression of Cx31.1 would decrease during NSCLC development. In cancer, the role of autophagy is context-dependent. It can be tumour suppressive through the elimination of oncogenic protein substrates, toxic unfolded proteins and damaged organelles. Alternatively, it can be tumour promoting in established cancers through autophagy-mediated intracellular recycling that provides substrates for metabolism [34]. Autophagy could be activated in more advanced stages of cancer to guarantee survival of cancer cells under extreme conditions, such as the restricted access of cells located in the inner areas of solid tumours to nutrients [35]. The degradation of Cx31.1 might ultimately lead to various tumour-suppressing effects. Once the autophagy is induced during the progression of NSCLC cells, the expression of Cx31.1 would decrease, which might contribute to the even worse phenotype of the cancer cells.

In conclusion, we found both ubiquitin–proteasome system (UPS) and autophagy participate in Cx31.1 degradation. Cx31.1 could interact with clathrin, and clathrin might involve in the autophagy of Cx31.1. It also shed a light on the mechanism of degradation pathway of other connexin family members.

## Supporting information

**Figure S1** (I) The knockdown efficiency of siRNA for ATG5. Cx31.1-EGFP-H1299 cells were transiently transfected with siATG5 for 48 hrs, cell lysates were then blotted with antibody against ATG5.

**Figure S2** CLQ treatment increased level of Cx31.1-EGFP.
