# Supplementary figures and images for: Connexin 31.1 degradation requires the Clathrin-mediated autophagy in NSCLC cell H1299
Source: J Cell Mol Med. 2014 Nov 11;19(1):257–64. doi: 10.1111/jcmm.12470 (PMC4288368; doi:10.1111/jcmm.12470)

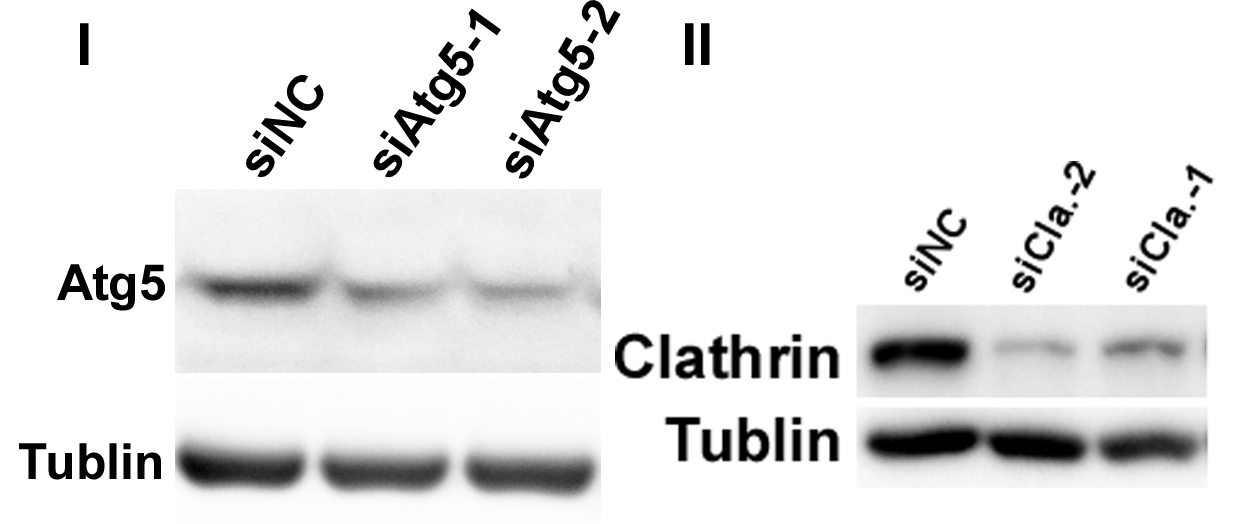

Supplement: Supplementary file 1 — Figure S1 (I) The knockdown efficiency of siRNA for ATG5. Cx31.1-EGFP-H1299 cells were transiently transfected with siATG5 for 48 hrs, cell lysates were then blotted with antibody against ATG5. [file jcmm0019-0257-sd1.tif]

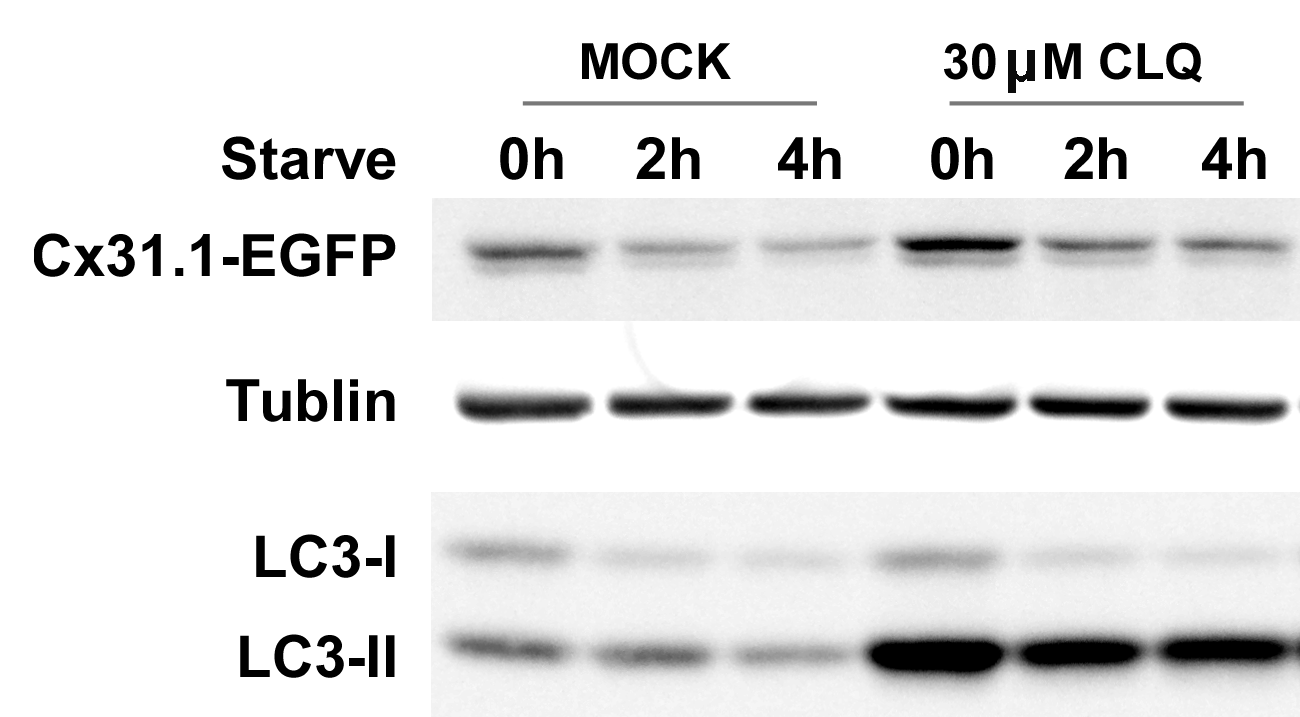

Supplement: Supplementary file 2 — Figure S2 CLQ treatment increased level of Cx31.1-EGFP. [file jcmm0019-0257-sd2.tif]

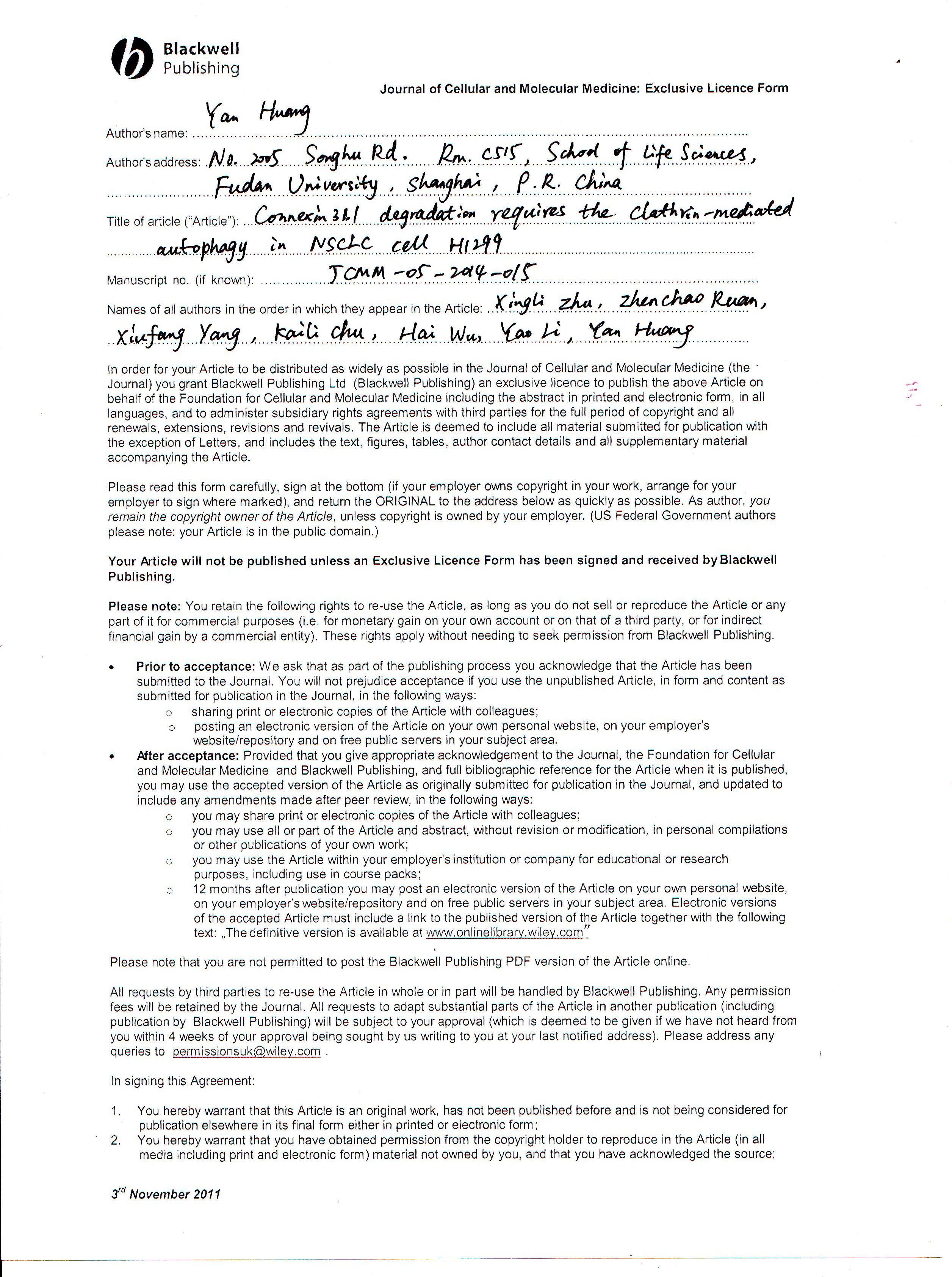

Supplement: Supplementary file 3 — Figure S2 CLQ treatment increased level of Cx31.1-EGFP. [file jcmm0019-0257-sd3.tif]

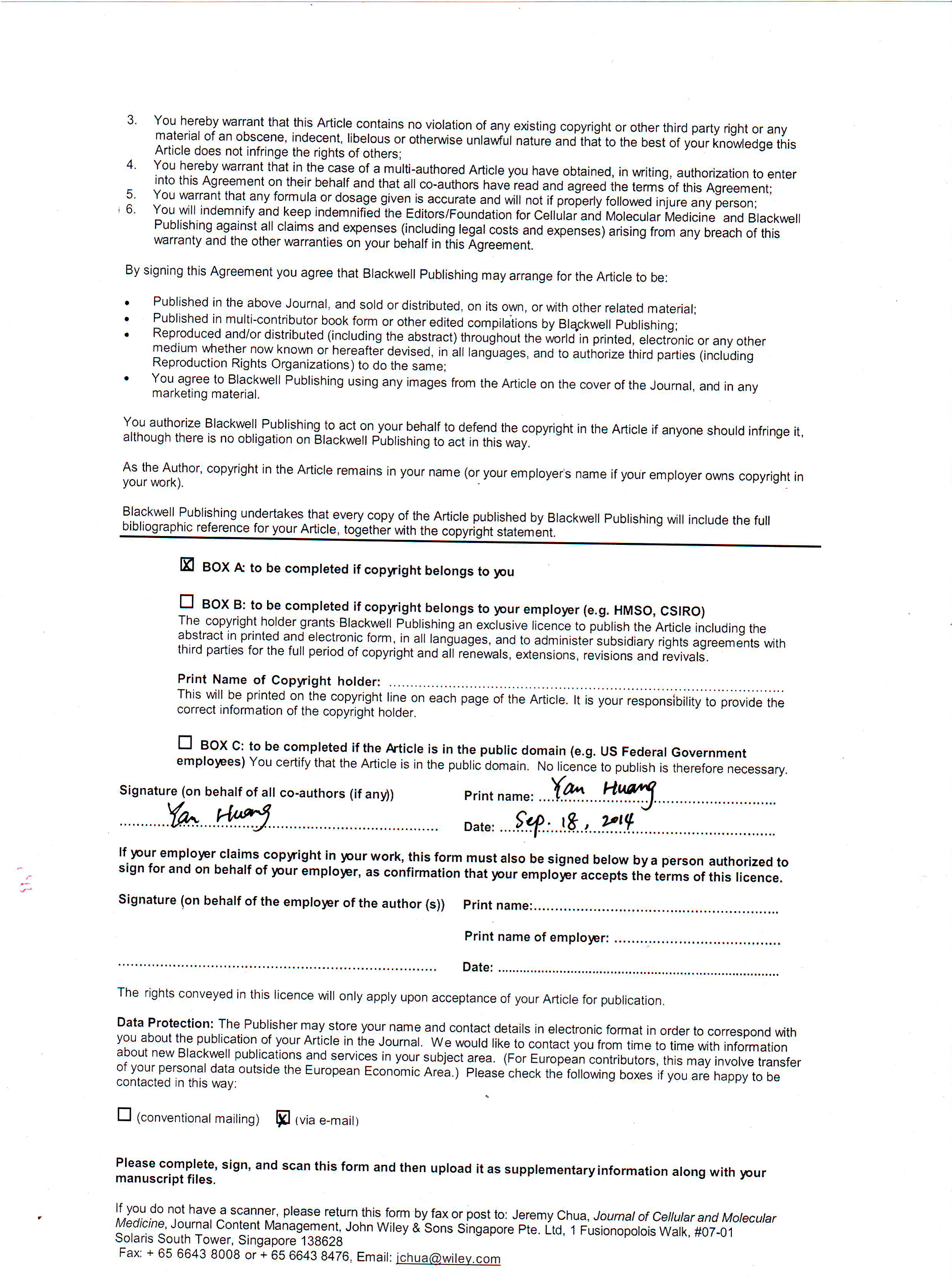

Supplement: Supplementary file 4 — Figure S2 CLQ treatment increased level of Cx31.1-EGFP. [file jcmm0019-0257-sd4.tif]
